# A Proteomic Atlas of Human Milk: Uncovering Milk Functional Components

**DOI:** 10.21203/rs.3.rs-10089470/v1

**Published:** 2026-08-25

**Authors:** Marco Agostino Deriu, Harry Zaverdas, Syed Taimoor Hussain Shah, Filip Stojceski, Konstantinos Panagiotopoulos, Konstantina Liontou, Seferina Mavroudi, María Carmen Durán-Ruiz, Foteini Kiskira, Athanasios Kalogeras, Christos Alexakos, Gianvito Grasso, Konstantinos Theofilatos

**Affiliations:** Politecnico di Torino; InSyBio PC; PolitoBIOMed Lab, Department of Mechanical and Aerospace Engineering, Politecnico di Torino, 10129 Turin, Italy; Dalle Molle Institute for Artificial Intelligence USI-SUPSI; PolitoBIOMed Lab, Department of Mechanical and Aerospace Engineering, Politecnico di Torino, 10129 Turin, Italy; InSyBio PC, Patras, 265 04, Greece; University of Patras; Biomedical Research and Innovation Institute of Cadiz (INiBICA), Cadiz, Spain; InSyBio PC, Patras, 265 04, Greece; Industrial Systems Institute; ATHENA RC; Dalle Molle Institute for Artificial Intelligence USI-SUPSI, Polo universitario Lugano-Campus Est, Lugano, Switzerland.; InSyBio PC

**Keywords:** Human Milk, Proteomics, Protein-Protein Interactions, Protein Regulatory Networks Cardiovascular Diseases

## Abstract

Human milk is a complex and highly organized biofluid whose molecular composition underpins neonatal nutrition, immune protection, and developmental regulation. Despite extensive proteomic profiling, its global organizational structure remains fragmented and poorly integrated. Here, we performed a large-scale meta-analysis of mass spectrometry–based proteomics datasets to construct a unified, queryable human milk proteome atlas for system-level interpretation. By harmonizing datasets through standardized preprocessing and normalization, we defined a core human milk proteome comprising the most recurrent and consistently detected proteins and peptides. An established machine-learning-based method was applied to predict binary protein–protein interactions from this core set, enabling reconstruction of a dense interaction network and moving beyond isolated protein catalogues toward a systems-level representation of milk organization. Network analysis revealed functional modules related to immune defence, vesicle-mediated secretion, and proteostatic control, while protein regulatory networks were also reconstructed. These results highlight the systems-level relevance of milk proteins and their potential role in maternal–infant regulation in health and disease. The integrative framework and web resource (http://atlas-galatea.insybio.com/) provide a foundation for comparative proteomics and network-based studies of the regulatory architecture of human milk.

Human milk represents one of nature–s most sophisticated and precisely calibrated nutritional systems, providing optimal nourishment for infant development during the critical early months of life. Beyond its role as a nutrient source, human milk functions as a structured biological system delivering coordinated immune, metabolic, and developmental signals to the neonate [1].

The composition of human milk demonstrates extraordinary complexity, containing a wide range of interacting molecular constituents [2], that work synergistically to support infant growth and development. Rather than forming a static mixture, human milk shows dynamic variability influenced by maternal physiology, environmental factors, and lactation stage, indicating a feeding system with context-dependent and adaptive organisation and reflecting millions of years of evolutionary refinement [3]. The intricate balance of macronutrients, micronutrients, bioactive compounds, and immunological factors within human milk, influences not only immediate nutritional outcomes but also establishes foundational patterns for immune system development [4], cognitive function [5], and metabolic reprogramming [6]. Together, these observations imply that human milk functions as an integrated molecular system where components are arranged in coordinated structures rather than operating independently. Such coordinated organisation suggests that milk transmits structured regulatory information at the maternal–infant interface.

Human milk oligosaccharides (HMOs), a key component of human milk, are the third most abundant solid component of breast milk (after lactose and fat), exemplifying the networked nature of milk’s biology [7]. Over 200 distinct HMO structures are known, synthesized in the mammary gland via complex enzymatic pathways. Recent systems biology approaches have elucidated the biosynthetic network of HMOs in unprecedented detail. For example, Kellman et al. [8] integrated glycomics and mammary gland transcriptomics to construct an HMO biosynthesis network and predict the specific glycosyltransferase enzymes involved. In a complementary in silico study, McDonald et al. [7] demonstrated that a minimal set of 11 enzymatic activities could generate the vast majority of identified HMO structures, thereby mapping a reaction network covering most of the HMO glycome. Beyond biosynthesis, HMOs engage in molecular interaction networks with microbes and the immune system. HMOs are indigestible by the infant but serve as selective substrates (“prebiotics”) for beneficial gut bacteria (notably Bifidobacterium and Lactobacillus species), thereby shaping the infant gut microbiota. Through these networked interactions, HMOs contribute to protecting infants from intestinal disorders (like necrotizing enterocolitis) and guide the development of the immune system [8]. Collectively, these studies show how a single class of milk components can be seen as part of a coordinated biosynthetic and interaction network, rather than as isolated bioactive molecules.

Proteins are the central functional layer within human milk contributing to infant nutrition, weight gain control, immune protection, microbiome development, and infant growth [1]. Proteomic analysis techniques have been developed to handle the complexity and dynamic range of milk proteins, implementing sample selection and fractionation techniques to detect low-abundance yet functionally critical proteins [9–11]. Research focus is centered on exploring temporal changes in the milk proteome across lactation stages [12], comparing milk from different species to pinpoint evolutionary differences and species-specific functions [13], investigating milk proteins’ glycosylation patterns [14], their role in microbial colonization and immune stimulation [15], and -importantly- understanding how milk proteomes respond to maternal conditions and disease [16].

However, in contrast to the increasingly resolved network architecture of HMOs, the protein layer of human milk remains comparatively fragmented across studies, lacking a unified systems-level reconstruction of its interaction and regulatory organization. Advances in liquid chromatography–mass spectrometry (LC–MS/MS) and quantitative proteomics have enabled increasingly comprehensive characterization of the human milk proteome, capturing both abundant structural proteins and low-abundance bioactive factors [17] [18] [19] [20] [21] [22] [23] [24] [25] [26] [27] [28]. Proteomics analyses on human milk have been employed to characterise whey proteins, glycoproteins, extracellular vesicle components, and species-specific peptides, as well as to explore temporal variation across lactation and responses to maternal physiological or pathological conditions [29] [30] [31] [32] [33] [34] [35]. Moreover, differences between human milk and bovine-based formula milk have been studied through proteomics, leading to the identification of hundreds of human-specific milk peptides [36]. Two notable studies have used an abundant set of milk samples derived from the CHILD Cohort Study [37], which were used for unravelling network associations of proteins, peptides, and between them, and for detecting relations between the allergy status of mother and child through the proteome differences in allergen abundances [38]. The above mentioned studies have substantially expanded the catalog of identified milk proteins and peptides and highlighted their functional diversity. However, most investigations have remained dataset-specific or functionally focused, providing detailed compositional insights without reconstructing an integrated systems-level architecture of protein interactions [39] [19].

Recent large-scale proteomics and peptidomics studies have revealed that milk proteins form distinct co-expression and interaction networks reflecting their origins and functions. Dekker et al. [40] applied network inference approaches to uncover tightly connected modules of proteins and peptides. These modules corresponded to shared secretory pathways, proteolytic processing events, and coordinated immune-related functions. Such findings suggest that certain milk proteins are organized into functional clusters rather than existing as independent units, reflecting shared biological origins (e.g., systemic transfer versus mammary synthesis) or hierarchical processing relationships, such as precursor–fragment cascades.

Using weighted correlation network analysis (WGCNA) [41], studies have delineated modules of co-varying milk proteins and peptides and linked them to maternal and infant attributes [42]. These modules reflect coordinated regulation across lactation stages and physiological contexts. Such network analyses of the milk proteome and peptidome not only map the interactions within milk (e.g., a lactoferrin-lactoferricin peptide network indicating enzyme cleavage of lactoferrin) but also provide biological context; for instance, the presence of a tightly connected cluster of immunoglobulins, lactoferrin, and lysozyme underscores a concerted immune defence role. Collectively, these studies indicate that the human milk proteome exhibits modular organization and coordinated regulation [40]; however, these reconstructions remain largely dataset-specific and do not yet provide a unified, systems-level interaction and regulatory architecture.

Although human milk proteomics is expanding quickly and modular co-expression analyses are emerging, the field still faces challenges such as fragmented datasets, varied preprocessing methods, and a lack of a unified interaction-resolved framework. As a consequence, the global architecture of protein–protein interactions and regulatory hierarchies within human milk remains incompletely defined. To tackle this challenge, we adopted a large-scale integrative approach that standardizes publicly available MS-based proteomics datasets through preprocessing, normalization, and filtering. This allows for comparison across studies and helps define a reliable core human milk proteome. From this core set, we reconstructed a protein–protein interaction landscape using a machine-learning–based predictive framework, generating a dense interaction network that substantially expands beyond existing curated repositories. This reconstruction revealed a highly interconnected modular structure in which immune-related proteins, including immunoglobulin components, emerge as central regulatory nodes. We further reconstructed a human milk interaction-derived network architecture and performed comparative analyses with established cardiometabolic regulatory networks, identifying shared central hubs such as IGHA1, IGKC, and IGHG1, and implying potentially therapeutic components of human milk.

Finally, selected high-centrality interaction pairs were modelled by means of protein–protein docking and subjected to molecular dynamics (MD) simulations to assess interface stability and conformational behaviour, thereby integrating atomistic structural dynamics into the reconstructed systems-level interaction framework.

## Results

**Figure 1:**
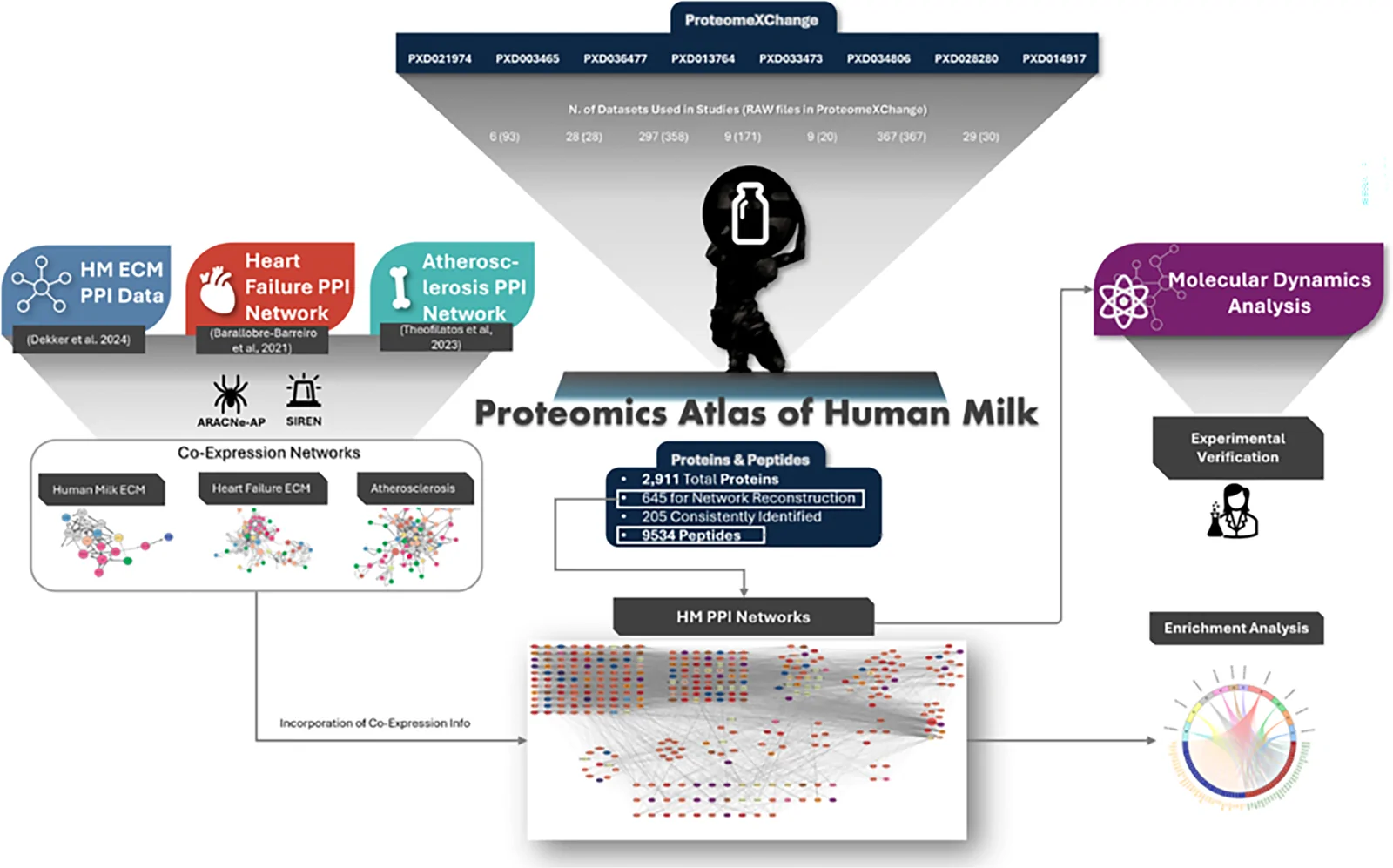
Overview of meta-analysis of publicly available proteomics data of human milk. Overall workflow for proteomics data analysis, regulatory and Protein-Protein Interaction network reconstruction, and Molecular Dynamics Analysis of selected interactions, which led to the establishment of the Proteomics Atlas of Human Milk.

The overall computational workflow for the meta-analysis of human milk MS data is presented in Fig. 1, including data mining from ProteomeXchange, network reconstruction and analysis, network comparison with networks related to cardiovascular diseases and additional Molecular Dynamics analysis/validation of selected PPIs from the reconstructed network.

The results are organized to progressively describe the molecular organization of human milk, moving from the characterization of its integrated proteome and the identification of a core proteomic set, to the analysis of protein associations and functional modules, and finally placing these molecular relationships in a broader biological context through cross-network comparisons and structural exploration of selected interactions.

### The human milk proteome and its core components

Large-scale reanalysis of publicly available MS datasets enabled the reconstruction of a unified human milk proteome and the identification of its most consistently represented components across studies.

After retrieving selected ProteomeXchange datasets described in Supplementary Table 1, and re-analyzing them with MaxQuant and the latest human protein library, we ended up with a filtered list of 2,911 proteins. By keeping those quantified in at least 20% of the samples across all datasets, a filtered list of 645 IDs was created, which constitutes the reported “core” human milk proteome. The GO Term enrichment analysis of the full list of 2,911 proteins composing the human milk proteome (Supplementary Fig. 1) showed statistically significant enrichment in translation processes and their involvement in the formation of cytosolic ribosomes (top 3 GO Terms: ‘cytosolic ribosome’/ QValue 1.39e^− 44^/ FE 5.68, ‘cytoplasmic translation’/ QValue 6.32e^− 44^/ FE 5.67, and ‘translation’/ QValue 8.00e^− 24^/ FE 2.96). In contrast, the filtered core proteome was predominantly enriched in immunological processes, while the filtered list was mostly enriched in immunological processes such as antigen binding, immune response and in the formation of immunoglobulin complexes (top 3 GO Terms: ‘antigen binding’/ QValue 1.11e^− 22^/ FE 8.17, ‘immunoglobulin complex’/ QValue 2.53e^− 19^/ FE 6.40, and ‘immune response’/ QValue 5.54e^− 15^/ FE 3.32), suggesting that the recurrent components of human milk are strongly associated with immune-related functions.

### The human milk PPI network and characteristics of protein functional clusters

**Figure 2:**
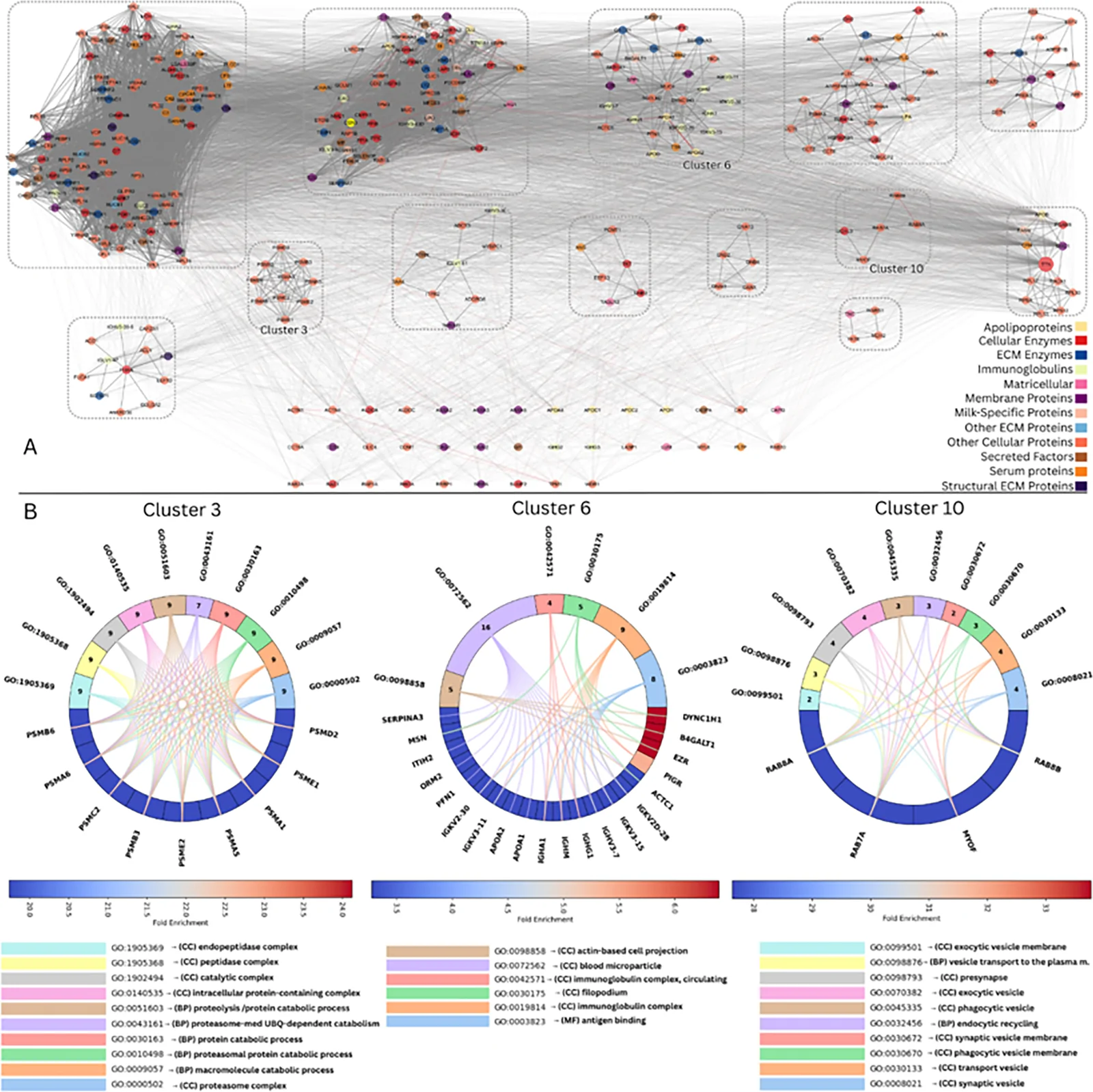
Reconstructed Protein-Protein Interaction Network of Human Milk. A) PPI network of Human Milk Proteins visualized with Cytoscape. Edge width is analogous to the corresponding edge weight in the same network. Node size is analogous to the Betweenness Centrality of the node, while node color corresponds to the protein groups according to the legend on the bottom right. Clusters are enclosed in grey dotted lines. The unclustered nodes are the ones involved in the HM Co-Expression Network’s edges, while the rest were filtered out. B) Circular graphs of the GO Term enrichment analysis results of the 3 top-ranked clusters based on the Silhouette-Coefficient. On the bottom half of each circle are the significantly enriched Genes of each cluster, ranked from left to right with ascending Fold Enrichment values (also colored from blue to red, respectively). Only Genes with adjusted p-value<0.05 are depicted in the graphs. GO Term clusters are present on the top half of the circle with distinct colors that are mapped below the graph to the GO Term description. Inside each GO term cluster cell, the number of Genes involved in the cluster is noted.

From the filtered list of 645 IDs, 207,690 potential binary combinations were created. Among these possible pairs, 12,550 PPIs were identified as positive by applying the previously established EOA method [43], forming the basis of the human milk protein interaction network analysed in this study. From these positive interactions, FASN-YWHAZ, LTF-CEL, and IGHG1-FASN were the top 3 milk-specific interactions in terms of Probability Score and Predicted Affinity. To explore the internal organization of the interaction network, clustering analysis was performed, identifying groups of proteins with shared topological and functional properties. The results of the network reconstruction, along with the top 3 clusters’ GO Term enrichment plots, are presented in Fig. 2. Proteins were grouped into functional and topological categories using an expanded version of the matrisomeDB to consider milk-specific proteins and cellular protein groups.

Functional enrichment analysis on the most central clusters (Clusters 3, 6, and 10, Fig. 2B) resulted in distinct functional enrichment patterns among the clustered proteins. These clusters highlight different functional contexts represented within the human milk proteome. Cluster 3 is highly enriched for proteasome-related processes (top 3 GO Terms: ‘proteasome complex’/ P-value 1.83e^− 12^/ FE 21.93, ‘peptidase complex’/ P-value 1.83e^− 12^/ FE 20.47, and ‘endopeptidase complex’/ P-value 1.83e^− 12^/ FE 20.47), including the proteasome core complex and endopeptidase activity, indicating potential involvement in protein degradation pathways. Cluster 6 shows significant enrichment in immune-related functions, particularly the immunoglobulin binding, suggesting a role in antigen processing and presentation (top 3 GO Terms: ‘blood microparticle’/ P-value 0.00013/ FE 3.27, ‘immunoglobulin complex’/ P-value 0.00034/ FE 5.42, and ‘actin-based cell projection’/ P-value 0.014/ FE 6.93). In contrast, Cluster 10 is predominantly associated with vesicle-mediated transport and membrane organization, with enrichment in exocytic vesicles, plasma membrane transport, and synaptic vesicle components (top 3 GO Terms: ‘synaptic vesicle’/ P-value 0.00012/ FE 30.7, ‘exocytic vesicle’/ P-value 0.00018/ FE 24.56, and ‘presynapse’/ P-value 0.00079/ FE 16.37). Supplementary Fig. 2 presents the functional enrichment analysis of all the clusters that had at least one significantly enriched (adjusted p-value < 0.05) GO Term cluster. Based on the protein groups enrichment results, these clusters do not comprise a specific protein group, but are rather generally associated as cellular proteins, which is more apparent in Clusters 3 and 10 (significant enrichment in the ‘Other Cellular Proteins’ group, with P-value 0.0006/ FE 2.07 and P-value 0.02/ FE 2.07, respectively). However, a subgroup of genes comprising Cluster 6 is found to be significantly (P-value 0.00015/ FE 4.23) involved in the Immunoglobulins protein group. The results of the Protein Groups enrichment results for the top-ranked clusters are presented in Supplementary Fig. 3. An overview of all the unclustered nodes can be found in Supplementary Fig. 4.

### Functional associations within the human milk proteome

To explore how proteins within the human milk proteome are associated across samples, we re-analysed correlation patterns among the proteins identified in the dataset from Dekker et al. [38], using the regulatory network reconstruction pipeline described in the Methods section. In this work, Dekker et al. described coordinated protein associations in human milk, with a particular focus on extracellular matrix (ECM) components. In agreement with these observations, our analysis revealed consistent association patterns among several proteins detected in our dataset, including HSPG2 (Perlecan), COL14A1, SERPINA1, and immunoglobulins (e.g., IGHA1, IGHG2, IGKC), which are highly abundant in human milk. The numerical values represent correlation coefficients between protein expression levels and their accompanying p-values, which indicate statistical significance. For instance, a strong correlation like HSPG2-COL14A1 = 0.7129 with a p-value ≈ 8.49E-11, indicates a strong and statistically significant co-variation in their abundance profiles across samples. These associations highlight potential functional relationships between ECM components and immune-related proteins in human milk, suggesting their possible involvement in neonatal development, immune regulation, and gut epithelial support. The consistent involvement of immune-related proteins (e.g., IGKC, IGHG1, IGHA1) in strong correlations with ECM components suggests the presence of coordinated molecular patterns linking structural and immune-associated proteins within the milk proteome.

### Shared hubs across distinct biological protein networks

Given that human milk is a secreted biofluid derived from maternal physiology, it is relevant to examine the extent to which its protein composition reflects broader molecular processes associated with the maternal organism. To address this, we compared the topology of the human milk network with previously published atherosclerotic [44] and ischemic heart failure-related[45] protein regulatory networks, with all 3 of them reconstructed using the exact same computational framework described in the Methods section, in order to identify recurrent central proteins and shared topological features.

The atherosclerosis-related network included proteins such as RAB6A, PCBP1, APOD, and COPE, together with proteins associated with mitochondrial function, oxidative stress, and extracellular matrix remodelling. The heart-failure network included extracellular matrix-related proteins and associated factors, spanning structural ECM components (e.g., COL6A1, LAMC1, LAMA5), immune-related proteins (e.g., IGHG1, IGHG3, CFH), and modulators of inflammation and fibrosis (e.g., CLU, POSTN, PRELP). As reported in that study, ECM proteins such as collagens (COL6A1/2/3), fibronectin (FN1), and decorin (DCN) were among the network components, together with complement- and immunoglobulin-related proteins including CFH, IGHG1, IGHA1, and IGKC, as well as proteins such as APOE, CLU, VCAN, ABI3BP, and ASPN.

To identify common hubs across systems, we performed a comparative network centrality analysis between the human milk and the heart disease PPI networks using the PageRank algorithm [46]. The PageRank score (PR) quantifies relative influence of a protein within the network topology, where higher values indicate nodes that sit on many well-connected paths [47]. Among the shared central proteins, IGLC2 (Immunoglobulin Lambda Constant 2) showed a PageRank score of 1.0 in the human milk network and was also highly ranked in the atherosclerosis network. Its centrality across both contexts is consistent with its established role in the constant region of immunoglobulin light chains and, more broadly, in adaptive immune function, including antibody production and mucosal immunity in milk [48], as well as inflammatory responses reported in atherosclerotic lesions [49–51]. Similarly, SERPINA1 (Alpha-1-antitrypsin) displayed a high PageRank in both networks, consistent with its known function as a regulatory anti-protease involved in tissue protection and modulation of proteolytic damage [52], and suggesting relevance to both milk-associated immune-protection and protease-driven matrix remodelling in atherosclerosis. Another shared hub was IGKC (Immunoglobulin Kappa Constant), with a PageRank of 0.87 in the milk network and a strong presence in the atherosclerosis network, further supporting the recurrence of immunoglobulin-related proteins among central nodes across the analysed networks. Additional proteins such as APOA1, HP (Haptoglobin), and APCS also showed significant centrality in both networks, supported by low p-values and false discovery rates. APOA1, a key lipid transport and anti-inflammatory protein [53], has recognised roles in milk-associated metabolic support [54, 55] and in cholesterol metabolism and plaque-related processes in atherosclerosis [56]. HP is involved in oxidative stress management and has been linked to endothelial protection in atherosclerosis [57, 58], while its presence in the milk network may also be consistent with broader host-defence functions. Proteins such as COL18A1, FN1, and CLU were also highly ranked in both networks and are associated with extracellular matrix remodelling [59], lipid metabolism [60], and tissue homeostasis [61], indicating recurrence of these functional themes across the compared network topologies.

In the ischemic heart failure network reconstructed from proteomic data of failing heart tissue [90], CFH (Complement Factor H) was among the most influential nodes and also showed a dominant role in the human milk network, with a PageRank score of 1.0 in the heart failure network. This overlap was supported by highly significant p-values (≈ 0.0005) and false discovery rates (FDR < 0.001) in the comparison between the two networks. CFH is known to participate in immune regulation, particularly through complement pathway inhibition and inflammation resolution, both in cardiovascular settings [62] and in human milk, including reported functional interplay with lactoferrin [63]. Several additional proteins also showed high PageRank scores in both networks, indicating recurring centrality across distinct biological contexts. FGB (Fibrinogen beta chain) and PRELP (Prolargin), for example, scored 0.79 and 0.76 in the heart failure network, respectively, and were also highly ranked in human milk. Their recurrence is consistent with shared themes related to coagulation, extracellular matrix regulation, and tissue remodelling. FGB has been linked to vascular injury responses [64] and immune activation [65], whereas PRELP contributes to connective tissue integrity [66] and extracellular matrix structural organisation [67]. ABI3BP and OGN also ranked among the top proteins. ABI3BP has been associated with cellular senescence, extracellular remodelling, and cardiovascular disease [68], while OGN (Osteoglycin) contributes to collagen fibril formation [69] and matrix mineralisation [70]. Overall, the recurrence of these proteins among central nodes suggests that extracellular matrix- and immune-related components represent shared topological features across the analysed networks.

To further explore common structural features across networks, we performed a comparative clustering analysis across the three MCL-clustered biological networks (atherosclerosis, human milk, and heart failure) to identify conserved proteins consistently grouped within biologically significant clusters, defined here as clusters containing at least five proteins.

**Figure 3:**
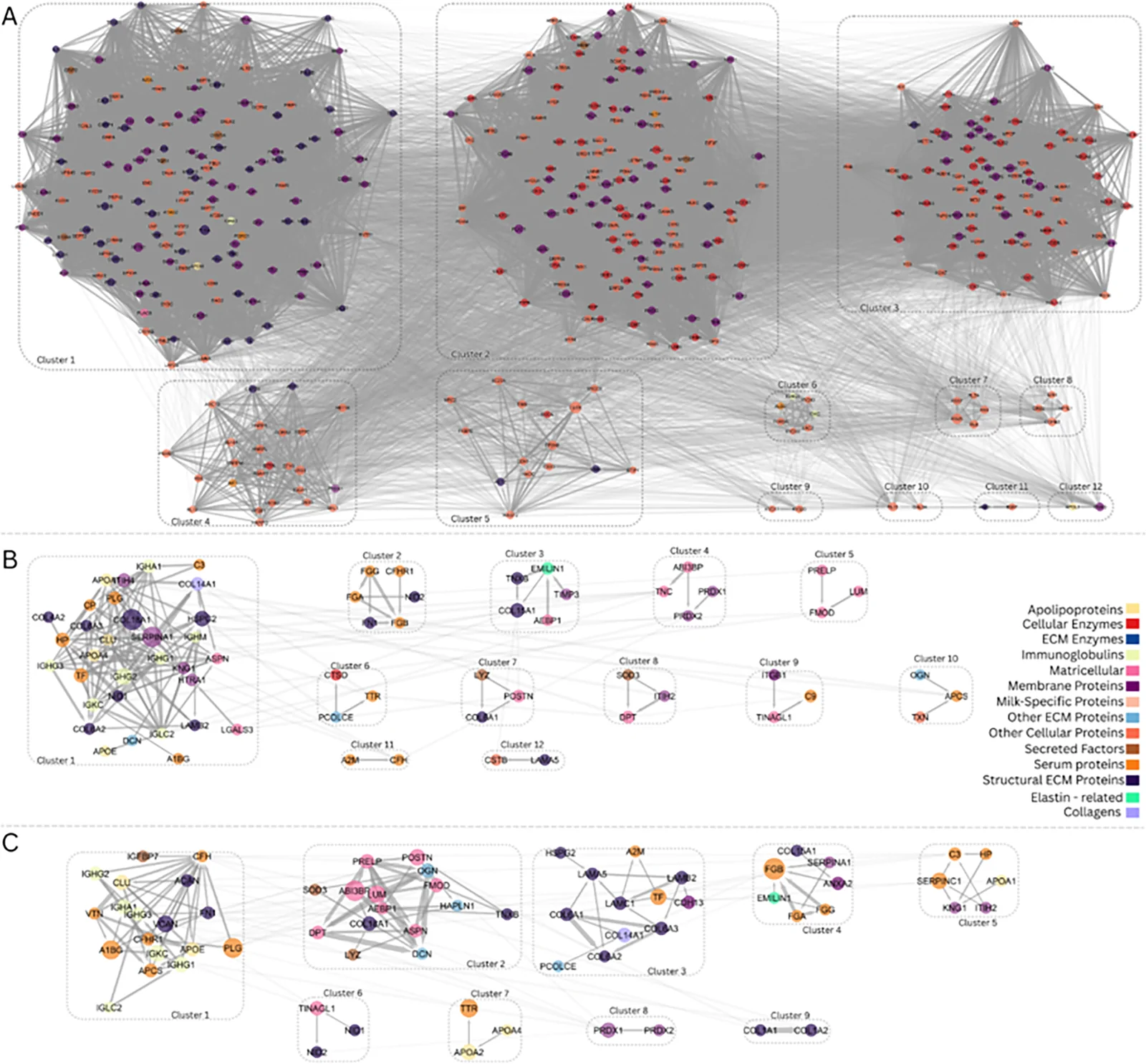
Human Milk and Heart Disease-related protein regulatory networks. A) Human Milk (top 12 clusters), B) Atherosclerosis, C) Heart Failure. Edge width is analogous to the corresponding Mutual Information (MI) index of the regulatory interaction, computed from ARACNe-AP reconstruction. Node size is analogous to the Betweenness Centrality of the node, while node color corresponds to the protein groups according to the legend on the middle-right. Clusters are enclosed in grey dotted lines. The unclustered nodes were filtered out.

**Figure 4:**
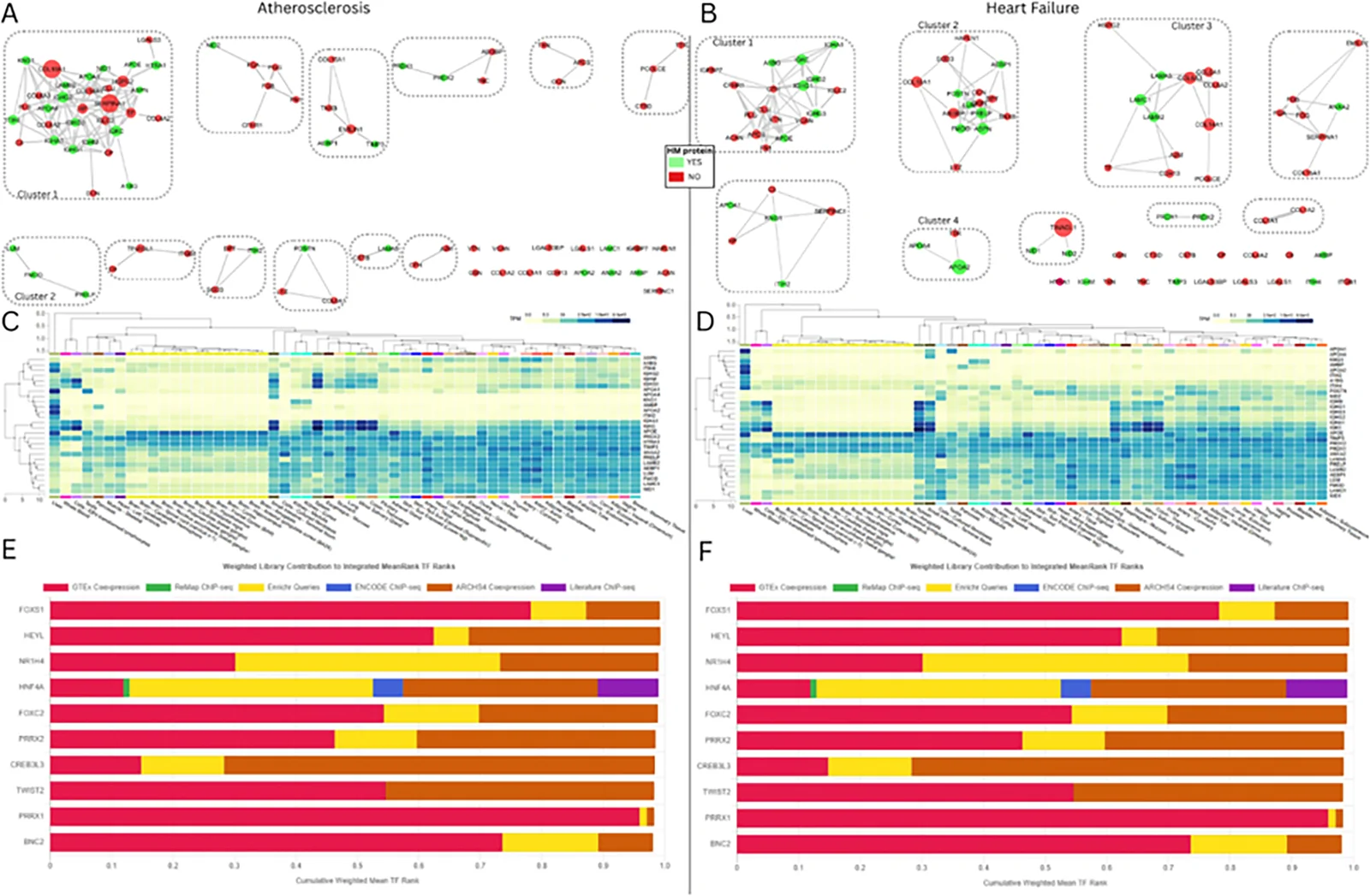
Regulatory network, tissue expression, and transcriptional enrichment of signatures shared between the human milk (HM) proteome and cardiovascular disease tissues. Regulatory networks are derived from the interactions of the common protein signatures of the HM regulatory network and (A) the atherosclerosis or (B) heart failure network. Green color represents the common proteins between HM and the respective network, while red represents the non-common ones that are interactors in these networks. GTEx-based tissue expression profiles of proteins (C) shared between human milk and atherosclerosis signatures, and (D) shared between human milk and heart failure signatures. Transcription factor enrichment analysis of proteins shared between (E) human milk and atherosclerosis signatures and (F) human milk and heart failure signatures, using the ChEA3 platform.

In addition, as shown in Fig. 4, HM proteome and cardiovascular disease signatures frequently occupy hub or bridge positions (Fig. 4A and 4B). More specifically, of particular interest are Clusters 1 and 2 in the HM-Atherosclerosis subfigure. Cluster 1 exhibits a subset of immunoglobulin genes (IGHG1, IGHG2, IGHG3, IGKC) with high centrality scores (Betweeness Centrality 0.36–0.46) that are also common nodes between the 3 studied regulatory networks (Fig. 3). Cluster 2 presents a gene group, all of which are common signatures between HM and atherosclerosis. In this, PRELP and LUM genes are found to regulate FMOD. In the HM-Heart Failure subfigure, a similarly high centrality (Betweeness Centrality 0.10–0.43) of the same immunoglobulin genes is found on Cluster 1. In Cluster 2, the triplet of FMOD-PRELP-LUM occupies central positions (max. Betweeness Centrality 0.49), with the same directionality, while in Cluster 3 the same can be said about another triplet of laminin genes (LAMA5, LAMC1, LAMB2, Betweeness Centrality 0.20–0.53) that is indirectly co-regulated with a subset of collagen genes in the same cluster (COL6A1, COL6A2, COL6A3, COL14A1).

Expression summaries for the two overlap sets of signatures (HM-Atherosclerosis and HM-Heart Failure) demonstrate broad distribution across several tissues in the GTEx analysis (Fig. 4C and 4D). A subset of HM-Atherosclerosis common genes (IGHA1, IGKC, APOE, PRDX2, HTRA1, TIMP3, ANXA2, PRELP, LAMB2, AEBP1, LUM, FMOD, LAMC1, NID1) exhibits high TPM scores in many tissues, spanning the digestive, cardiovascular, endocrine, reproductive, and integumentary systems, while IGHA1, IGKC, APOE, PRDX2, and HTRA1 are also in brain tissues. The majority of these genes (except IGHA1 and HTRA1) that are expressed in all GTEx-available tissues are also common between the HM-Heart Failure and HM-Atherosclerosis networks.

Complementing the network and expression views, transcription-factor (TF) enrichment of the shared signatures pinpoints upstream regulators confirmed across multiple libraries through the ChEA3 platform (Fig. 4E and 4F). Specifically, the TFs found enriched in both common networks were FOXS1, HEYL, NR1H4, HNF4A, FOXC2, PRRX2, CREB3L3, TWIST2, PRRX1, and BNC2. Factors like FOXS1, FOXC2, TWIST2, and BNC2 suggest that the TFs of genes common to HM and cardiovascular diseases are likely linked to Epithelial-to-Mesenchymal Transition (EMT) or Tissue Fibrosis, while the inclusion of HNF4A, NR1H4, and CREB3L3 highlights a strong connection of said genes to liver metabolism (as also evident by the GTEx tissue expression analysis).

The meta-analysis of cardiovascular disease–associated proteins retained in the human milk regulatory network reveals a strong convergence into conserved regulatory modules. This convergence is consistently supported across all three analyses (regulatory network topology, GTEx-based tissue expression, and transcription factor enrichment) and highlights the predominance of immune-related and extracellular matrix–associated programs that are shared between human milk biology and cardiovascular disease pathology.

### Molecular Dynamics

**Figure 5:**
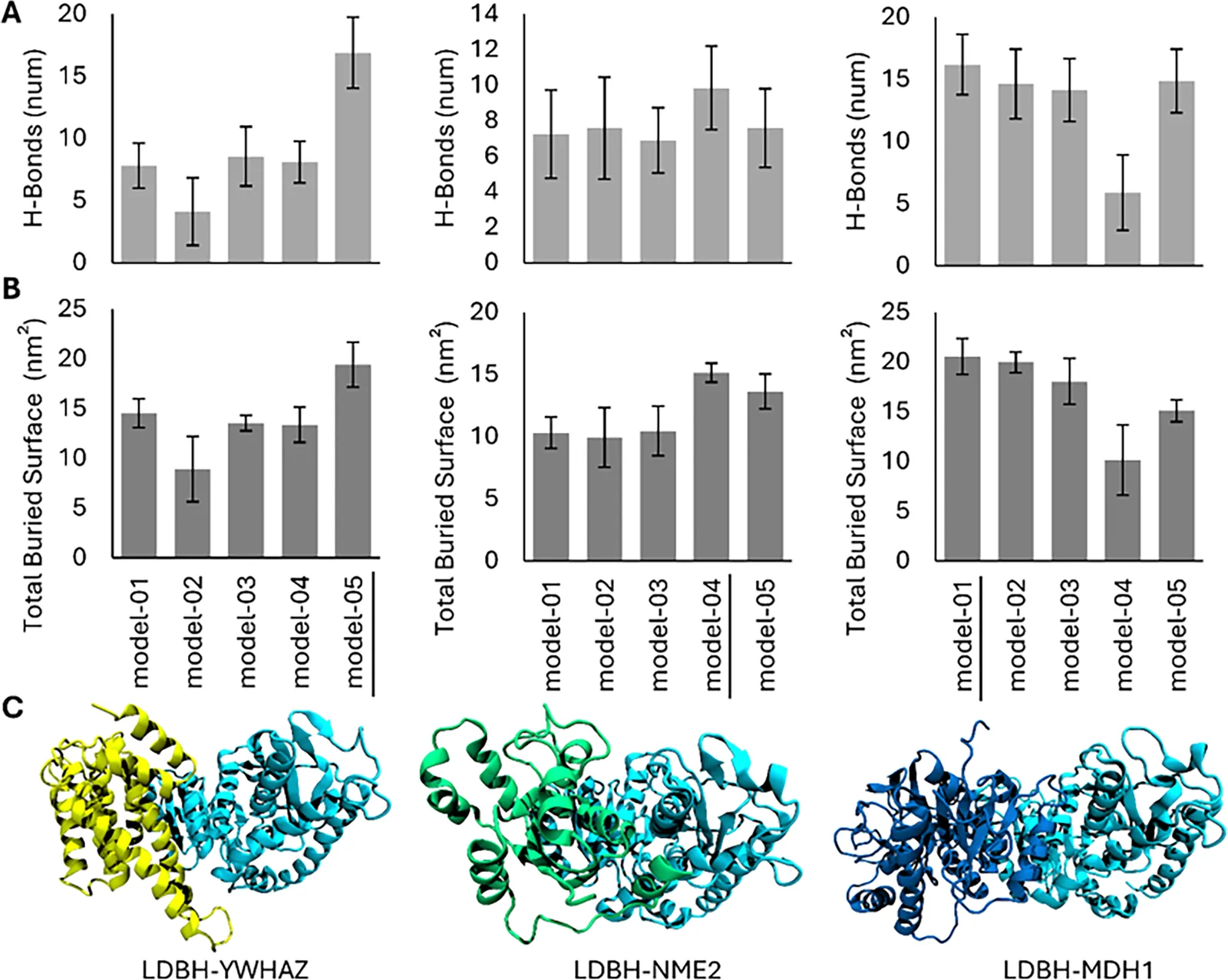
Hydrogen Bonds and Buried Surface Area calculations for each pose of the top-ranked Human Milk PPIs. A) Hydrogen bond analysis of the 5 docking models for each of the 3 selected PPI pairs: LDHB–YWHAZ, LDHB–NME2, and LDHB–MDH1. The results are reported as the average and standard deviation of the 3 replicas on each docking model. B) Total buried surface analysis of the five docking models for each of the three selected PPI pairs: LDHB–YWHAZ, LDHB–NME2, and LDHB–MDH1. The results are reported as average and standard deviation of the 3 replicas on each docking model. C) Qualitative representations of the 3 PPI pairs involved in the highest interacting models.

To elucidate the structural stability of the top-ranked PPIs identified by our ML-based pipeline, we performed all-atom MD simulations on three selected binary complexes, LDBH-YWHAZ, LDBH-NME2, and LDBH-MDH1. For each PPI pair, five distinct docking poses generated by HDOCKlite were subjected to 200 ns of simulation in three replicas. To quantify the interaction behaviour of these interactions, we analysed the trajectories of the last 20 ns, calculating the average number of hydrogen bonds (H-bonds) and the buried surface area (BSA) for each pose (Fig. 5A and 5B). This analysis allowed us to identify the most conformations with the highest H-bonds and BSA for each complex.

For the LDHB-YWHAZ complex, model 5 demonstrated the most favorable interaction behavior. This model maintained an average of 16.88 ± 2.88 H-bonds and a BSA of 19.41 ± 2.25 nm^2^. The high number of persistent H-bonds coupled with a substantial buried surface suggests a stable and potentially specific interaction between the glycolytic enzyme and the regulatory 14–3-3 protein. In the case of the LDHB-NME2 interaction, model 4 was identified as the one with the highest interaction metrics among the others. This complex exhibited an average of 9.83 ± 2.35 H-bonds and a BSA of 15.13 ± 0.77 nm^2^. Finally, the LDHB-MDH1 complex showed the strongest binding interface in model 1. This model showed an average of 16.18 ± 2.43 H-bonds and the largest BSA among the three complexes at 20.55 ± 1.81 nm^2^ (Fig. 5A). The extensive buried surface suggests that the interaction between LDHB and MDH1 involves a significant portion of their solvent-accessible surfaces.

Qualitative representations of the PPI pairs involved in the highest interacting models are provided in Fig. 5C, highlighting the spatial orientation of the interacting partners. The successful stabilization of these complexes in silico provides structural evidence supporting the biological plausibility of these PPIs within the human milk proteome. In addition, the root mean square deviation (rmsd) analyses of the top interacting PPIs are provided in Supplementary Fig. 5.

### Web platform of Proteomic Atlas of Human Milk

To facilitate researchers in the exploration of the human milk proteome, a dedicated web interface was developed that integrates quantitative proteomics data, predicted PPI and regulatory networks, and curated external annotations into a single queryable framework. The interface is structured to provide both protein-centric and network-centric information, capturing all the levels of analysis of the present study. The main functionalities (Supplementary Fig. 6) include a home page that serves as a central access point, allowing users to search for individual proteins and their interaction networks. This design enables switching between molecular-level information and system-level information in the HM proteome. The protein search module provides structured access to all proteins identified in the meta-analysis, as well as to their standardized identifiers, sequence information, dataset coverage, and functional classification, as well as direct links to UniProt, HUGO, and RefSeq databases. The resources section supports data availability efforts, providing direct downloads of all major outputs generated in the study, including comprehensive protein lists, filtered protein sets used for network inference, and MaxQuant-derived datasets harmonized across studies, enabling researchers to independently validate and reanalyze the generated data. Network-level exploration is supported both in the predicted PPI network and in the regulatory network. The global PPI network view (Supplementary Fig. 6D) further displays the proteins grouped according to functional categories and clustered based on network topology. This allows users to identify densely connected modules and assess the distribution of biological functions across the network. In parallel, the interface supports targeted subnetwork extraction (Supplementary Fig. 6E), enabling visualization of interaction neighborhoods for user-selected proteins, enabling hypothesis-driven analyses, such as examining the interaction context of specific groups of proteins of interest.

## Discussion

The present study aimed to meta-analyze existing mass spectrometry–based proteomics datasets of human milk by applying consistent preprocessing, normalization, filtering, and imputation workflows, ultimately generating a queryable repository. By further applying an ML–based pipeline to reconstruct protein–protein interaction (PPI) and clustering algorithms the goal was to detect key hubs of HM-related proteins and their interactions. The network relationships of these milk-specific proteins provided a framework for guiding future experimental validation of their functional roles and their involvement in already established molecular pathways. In addition, using the largest publicly available human milk proteomics dataset (Dekker et al.), a protein regulatory network was reconstructed and compared with atherosclerotic and heart tissue-related networks to identify disease-specific proteins and clusters. Based on the combination of protein centrality, statistical significance (p-value and False Discovery Rate), and PageRank (PR) score, the most prominent shared proteins between the human milk and atherosclerosis-related networks were IGLC2, SERPINA1, and IGKC, while CFH, FGB, and PRELP were shared between the human milk and heart tissue networks, and IGHA1, IGKC, and IGHG1 were common to all three networks. These results provided important insights into the molecular pathways mediated by human milk and established a foundation for exploring its potential therapeutic properties.

The MaxQuant-based preprocessing of the ProteomeXChange datasets led to the identification of 2,911 unique human milk proteins with a filtering criterion (≥ 20% identification across datasets), which restricted this to 645 consistently detected proteins, representing the “core” human milk proteome. It is worth noting the complexity of human milk across populations, which, coupled with the different sample conditions and software preprocessing configurations, restricts a consensus on the consistency of the HM proteome. An earlier study[71] trying to merge 38 publicly available proteomic datasets of human milk identified a similar number of 2,698 unique proteins, though this presented a significant shift from proteins identified in milk-derived EVs (1,963). Subsequently, the exponential development of proteomics through LC-MS/MS analysis changed the landscape of the HM proteome. The most recent work from Dekker et al[72, 73] has analyzed a thorough dataset of 300 mother-child dyads from the CHILD cohort (https://www.childstudy.ca), identifying 1,690 proteins that, upon filtering for consistency in the mother-child allergy groups, reached 687, which was surprisingly similar to the filtered network proteins in the present study (645). Even though different filters and different datasets/groups were used as input in MaxQuant, more than half of the filtered proteins (377) were common, indicating a core proteome that can be assessed and meta-analyzed for its biological significance.

The enrichment of translation- and ribosome-related GO terms in the full set of 2,911 proteins likely indicates involvement in secretion-related processes, which can be attributed to milk’s cellular origin (epithelial and immune cells). Specifically, the lactating mammary gland consists of branching ductal networks made up of epithelial cells that form extensive lobulo-alveolar clusters, serving as the site of milk production and secretion[74, 75], and subsequently, HM is expected to be enriched in proteins involved in this process. The filtered core proteome of 645 proteins shows a clear functional shift toward immune-related processes, in accordance with the known biological specificity of human milk in neonatal immune protection and passive immunity transfer[75, 76]. This enrichment in antigen binding, immunoglobulin complex formation, and immune response terms is well characterized in HM enrichment studies[77], but also underscores that consistency filtering helps narrow down functional proteins actively secreted into milk from background cellular components, helping identify HM’s core functions.

From these 645 proteins, 12,550 positive HM PPIs were identified using the EOA methodology, and were used as input for the network reconstruction process and for the clustering algorithms. From the positive interactions, FASN-YWHAZ, LTF-CEL, and IGHG1-FASN being the top 3 milk-specific interactions in terms of Probability Score and Predicted Affinity. The network clustering was performed using the MCODE algorithm for clustering based on a Silhouette Coefficient (SC) criterion (see relevant Supplementary Table), leading to a 645-node highly interconnected system. The clusters were individually assessed based on their per-cluster SC, and GO term enrichment analysis was performed to reveal the cluster-specific functions in comparison to all other network proteins. The most biologically significant clusters (in terms of GO term enrichments of adj. p-val < 0.05) appeared to be Clusters 3,6, and 10.

Cluster 3, enriched in proteasome and peptidase proteins, potentially reflects the proteostatic control mechanisms essential to secretory mammary epithelial cells[75]. During lactation, these cells synthesize and secrete large quantities of milk proteins via the exocytotic pathway, requiring strict protein folding and degradation systems to maintain secretion efficiency[78, 79]. Proteasome and peptidase activity help remove misfolded or excess proteins before secretion, thereby sustaining milk proteostasis. Consistent with this, the mammary gland possesses a regulated proteolytic system, including enzymes such as plasmin and cathepsin D, which manage protein turnover and peptide generation within milk[80, 81]. Cluster 3, being highly homogenous in milk-specific proteins (see Fig. 2), is likely a subset of the quality-control and degradation machinery that ensures protein homeostasis and prevents the accumulation of misfolded proteins in the secretory pathway. It is worth noting that two of the proteins in the cluster (PA28α and PA28β subunits, products of PSME1 and PSME2 genes) form the PA28 (or 11S) complex that characterizes the eukaryotic immunoproteasomes[82]. So far, to our knowledge, no studies have described the functionality of immunoproteasome in human milk and its interactions with other proteasomal proteins, which can lead to potentially novel findings about an HM-specific interplay between proteostatic control and HM’s innate immunity mechanisms.

Cluster 6 was found enriched in immunoglobulin binding and immune processes, which is expected, given the similar enrichment of the 645 proteins that were used as input to the network and the immunological abilities of HM that were described thoroughly. This cluster suggests a networked immune module possibly responsible for antigen presentation, antibody transport, or immune complex stabilization within milk. But further study is needed to unravel specific interactions of the antigen-binding/ immunoglobulin complex-specific proteins that form this cluster (IGKV2–30, IGKV3–11, IGHA1, IGHM, IGHG1, PIGR, IGHV3–7, IGKV3–15, IGKV2D-28) with others within it that have a high Fold Enrichment score and take part in actin-based cell projection and filopodium formation (MSN, EZR, B4GALT1, DYNC1H1, ACTC1). Filopodia are believed to promote viral infection and enhance cell-to-cell viral spread by increasing transmission speed, enabling immune evasion, helping viruses overcome physiological barriers, and enhancing the infectivity of target cells. These indirect associations between filopodia proteins and immunoglobulins could be the groundwork for further study of the immunological block of filopodia-based viral spread, which could also take place in HM.

Cluster 10 is another highly homogenous cluster, as 4/5 proteins are milk-specific and involved in vesicle-mediated transport or form exocytic vesicles, representing the well-characterized vesicle-driven secretion mechanisms of HM, which include exosomes or microvesicles, which are known carriers of bioactive molecules from mother to infant[75]. The existence of Ubiquitin carboxyl-terminal hydrolase isozyme L3 (UCHL3), a deubiquitination enzyme, in this interacting cluster could be further studied. Ubiquitination (and deubiquitination) is well known to regulate many steps in the secretory pathway, including cargo sorting, vesicle formation, and endocytosis/exocytosis[83]. Some studies have suggested a positive role of deubiquitination enzymes (DUBs) in cell signaling, as DUBs use their influence in cell architecture to promote cell signaling[84]. Further exploring DUBs such as UCHL3 in relation to signaling mechanisms, such as the EV-mediated one that is crucial in HM.

The network reconstruction of the HM-specific PPIs showcased a set of functionally coherent and homogeneous clusters that cover major themes of HM function, such as immune protection and EV formation. Each of these clusters represents a distinct but interconnected facet of human milk biology, from proteostatic regulation (Cluster 3) and immune defense mechanisms (Cluster 6) to EV-mediated secretion and trafficking (Cluster 10). Clusters 3, 6, and 10 underscore the robustness of the proposed methodology to identify biologically significant modules in the reconstructed network, and this consistency paves the way for further experimental study and meta-analysis of the clustered interactions, aimed at elucidating the molecular architecture of human milk and its role in maternal–infant immunological and metabolic communication.

The human milk protein regulatory network reveals a finely tuned system of molecular interactions that underpin the biological richness and functional complexity of this vital secretion. Given the scale and density of the human milk protein regulatory network (1459 nodes and 132962 edges), we prioritized Markov Clustering (MCL) over other algorithms as its flow-based expansion inflation procedure is well-suited to large, noisy biological interaction graphs, where communities correspond to regions of high random-walk retention. Unlike many heuristics, MCL has a single, interpretable knob, the inflation parameter, that directly controls cluster granularity and enables transparent, reproducible tuning. MCL has a long track record of strong performance on network clustering problems [85–87], and high-performance implementations (e.g., HipMCL[88]) demonstrate scalability to large numbers of nodes and edges, properties that match our network’s size and our need for well-separated modules. We benchmarked candidate inflations (≈ 1.5–3.0) and selected 2.7 as the settings that best balanced resolution and stability: it maximized graph modularity, yielded consistent partitions across bootstraps (high adjusted Rand index), and was robust to modest edge-weight perturbations. Practically, MCL is fast, memory-efficient, and produces non-overlapping, compact modules that are easy to interpret, making it an appropriate choice for downstream functional analyses of our ARACNe-AP/SIREN-derived network.

Using the ARACNe-AP pipeline followed by SIREN-based directionality assignment and MCL clustering (inflation rate 2.7), the network decomposes into tightly knit modules that reflect specific biological tasks. A key observation is the centrality of immunoglobulin-related proteins such as IGHA1, IGKC, and IGHG2, which consistently appear in dominant clusters and exhibit strong mutual interactions. These proteins form the backbone of passive immunity conferred to the neonate, highlighting the network–s primary focus on immune protection.

IGHA1 [89] was consistently identified in the biggest cluster across atherosclerosis, human milk, and heart failure networks. Its uniform clustering highlights its fundamental immunological role, likely involving mucosal immunity and antibody-mediated response, consistent with its known distribution in secretions and involvement in systemic inflammation.

IGHG1[90] and IGKC[91], were also highly central across human milk and cardiovascular tissues. IGHA1 is known to be correlated with multiple cardiovascular conditions, such as diabetic angiopathy, congestive heart failure, and hypertension, influencing vascular elasticity and heart contractility[92, 93]. Studies have also shown that the aortic valves of clonal hematopoiesis patients (shown to be linked with cardiovascular disorders[94]) contain higher transcription levels of IGHG1 than those without[95], and more interestingly, was shown to be upregulated in advanced-stage carotid atherosclerosis[96]. scRNA-seq and mRNA-seq differential expression analyses have shown upregulation of IGKC in endothelial cell populations in atherosclerotic plaques compared to normal tissue[97], while in microarray expression data from atheroma plaques showed it to be significantly downregulated[98], potentially indicating that the regulation of IGKC is correlated with atherosclerosis pathology. This triangulated cluster overlap reveals how immunoglobulin components retain strong modular integration regardless of the surrounding biological system, whether circulating in the vasculature during atherosclerotic stress, contributing to immune defense in milk, or participating in inflammatory cascades in heart failure. Their consistent inclusion in robust clusters strengthens their candidacy as core biomarkers or cross-condition immune mediators, highlighting the value of multi-network systems biology in uncovering conserved molecular scaffolds that underpin physiological resilience or disease propagation.

Equally prominent are clusters dominated by extracellular matrix (ECM) components, including COL14A1, FN1, and HSPG2, which play crucial roles in structural support and cellular adhesion. The co-localization of immune and ECM proteins within clusters suggests a coordinated mechanism of mucosal defence and tissue development, emphasizing the dual immuno-structural function of human milk. Clusters also encompass proteins involved in lipid transport, antimicrobial defence, and growth signalling, each representing a microdomain of co-regulated activity. The fine-grained clustering enabled by the high inflation parameter ensures that these functional units are distinctly resolved, providing an interpretable framework for understanding how diverse protein functions are compartmentalized within the milk proteome. These insights reinforce the idea that human milk is a highly organized biological fluid, where immune surveillance, nutrient transport, and developmental cues are orchestrated through a modular protein network.

Interestingly, the human milk network also contains several proteins that are classically associated with cardiovascular disease (CVD), suggesting a broader systemic relevance of milk-borne molecules. Comparative analyses with atherosclerosis and heart failure regulatory networks, reconstructed and analyzed with the same workflow, revealed overlapping high-centrality proteins, such as CFH, FN1, CLU, and APOA1, which retain influential positions across all three conditions. Their presence within major clusters in the milk network implies that these proteins are not only essential for infant health but may also be key regulators in systemic immune and inflammatory processes. For example, CFH (Complement Factor H), identified as the most central node in the heart failure network, also emerges prominently in the milk interactome, where it likely contributes to immune modulation and pathogen clearance at mucosal surfaces.

Similarly, FN1 (Fibronectin 1) and CLU (Clusterin) participate in clusters associated with tissue remodelling and lipid metabolism functions equally relevant to neonatal tissue development and vascular repair mechanisms in CVD. These shared proteins act as molecular bridges between physiological (milk-based) and pathological (CVD-related) environments, highlighting the functional plasticity of immune-ECM mediators. Their clustering within human milk reflects a sophisticated system where evolutionarily conserved proteins serve dual roles in both protection and structural regulation. The identification of such CVD-related hubs in milk reinforces the hypothesis that maternal milk not only nourishes but may also impart systemic resilience through the transfer of multifunctional proteins. This finding opens new avenues for exploring milk-derived components as potential biomarkers or therapeutic leads for cardiovascular conditions, as well as for understanding maternal-neonatal molecular crosstalk in health and disease.

The regulatory network meta-analysis (Fig. 4) further argues that proteins shared between the HM and cardiovascular disease tissue networks are organized into coherent modules. The localization of shared proteins within high-centrality regions of the network is an indication of conserved regulatory programs that are apparent in both maternal milk secretion and cardiovascular disease states. This theory is based on the assumption that hub and bridge nodes often exert significant influence over system behaviour[99]. At first, immunoglobulin-centered clusters are present in all regulatory networks, especially IGHA1, IGKC, and IGHG1, further linking HM immunity with chronic inflammatory processes characteristic of atherosclerosis and heart failure. In parallel, extracellular matrix–related modules, particularly the PRELP–LUM–FMOD axis and laminin–collagen subclusters, emerge as conserved features across the shared networks. These ECM proteins are regulators of collagen fibrillogenesis, tissue stiffness, and fibrotic remodelling, while being linked in cardiovascular pathology[100–104], and their apparent regulation in human milk suggests that similar molecular systems may guide intestinal maturation and tissue organization in the neonate. Importantly, this network-level convergence is independently validated by GTEx expression analyses, which show that shared regulatory genes are consistently expressed across cardiovascular, digestive, endocrine, and neural tissues, indicating systemic physiological relevance. TF enrichment further also shows that the shared genes are regulated by TF associated with epithelial-to-mesenchymal transition and fibrosis (FOXC2, TWIST2, PRRX1/2)[105–107], as well as metabolic and lipid regulatory pathways (HNF4A, NR1H4, CREB3L3)[108, 109], all of which are drivers of inflammatory remodelling and metabolic dysregulation in cardiovascular disease[110–112]. Summarizing, the regulatory network analyses support a model in which human milk may preserve immune and extracellular matrix regulatory systems that are also activated during cardiovascular pathology, providing a framework for understanding how milk-derived proteins may function as regulators that support neonatal development while also participating in inflammatory and remodelling processes later in life.

To complement the network-level findings, we performed MD simulations on top-scoring milk-specific PPIs to validate their structural stability and uncover mechanistic details that cannot be inferred from network analysis alone. Among the identified pairs, LDHB-centered interactions emerged as dominant among top scoring milk-specific PPIs. In particular, the LDHB–YWHAZ complex displayed strong stability, supported by extensive hydrogen bonding and a large buried surface area, suggesting a regulatory interaction between lactate metabolism and 14–3-3–mediated signalling[113]. Similarly, the LDHB–NME2 interaction is supported by existing experimental evidence, reinforcing the robustness of our computational predictions[114]. These findings indicate that key metabolic enzymes in human milk may participate in structured regulatory complexes, linking energy metabolism with broader signalling pathways. Additional analyses on these PPIs are provided in the Supplementary Discussion 1.

Several limitations of the proposed methodologies should be acknowledged. First, the meta-analysis of the ProteomeXChange datasets relied on integrating samples acquired under heterogeneous experimental conditions, and these methodological discrepancies likely introduced batch effects and platform-specific biases that could not be fully mitigated despite standardized preprocessing and normalization. Moreover, only proteins identified and quantified across ≥ 20% of the samples were included in downstream analyses, which may have excluded low abundance but biologically relevant proteins. Second, ML–based reconstruction of protein–protein interaction and regulatory networks provided valuable insights, but these computational networks are inherently limited by the incompleteness and potential false-positivity of available interaction repositories and the predictive nature of ML models; network edges do not reflect confirmed biochemical interactions, and experimental validation remains essential. Furthermore, the regulatory network reconstruction using ARACNe-AP and SIREN was based on proteomic abundance profiles, which capture correlation but not causality. Third, the current analysis did not include temporal or longitudinal dimensions of milk composition, which are known to vary across lactation stages and maternal physiological states. Future work involves expanding this framework through multi-omics integration, as recent systems biology initiatives, such as the Phoenix Study [115], and multi-omic milk network analyses by Chandran et al. [116] and others [117, 118], have demonstrated the value of integrating milk’s proteome with its metabolome, microbiome, and immunome to identify novel biomarkers predictive of infant immune development and disease risk.

Conclusively, this study represents the first large-scale meta-analysis of human milk proteomics datasets integrated through a standardized preprocessing and normalization framework and complemented by ML–based network reconstruction. By harmonizing raw human milk samples from 8 ProteomeXChange studies, we defined a robust “core” human milk proteome of 645 consistently quantified proteins. The applied EOA pipeline was able to predict 12,550 binary PPIs from these 645 proteins, with LDBH-YWHAZ, LDBH-NME2, and LDBH-MDH1 being the top-scoring milk-specific interactions in terms of probability score and predicted affinity. Their structural stability was characterized thoroughly with MD analysis, showcasing significant stabilization of the complexes and further confirming their biological significance. The critical role of LDHB in the HM function and its dysregulation in disease states serve as an indication of the importance of these interactions, whose further exploration may provide insights into novel therapeutic markers contained in the HM proteome. The reconstructed PPI network from these 12,550 PPIs further verified the key functional properties of human milk by showcasing distinct clusters enriched in immune defence, vesicle-mediated secretion, and proteostatic control terms. Furthermore, the protein regulatory networks of human milk, atherosclerosis, and heart failure highlighted proteins such as IGHA1, IGKC, and IGHG1 as central mediators that bridge milk-specific and cardiovascular-related molecular systems. These findings emphasize that human milk proteins not only play a role in neonatal immunity but also exhibit systemic biological relevance, potentially linking maternal and infant cardiovascular and immune health.

Finally, by incorporating these findings in a unified publicly available web interface, the aim was to enhance reproducibility and data reuse by providing direct access to all resources used in constructing the atlas. In this atlas, users can query for proteins and peptides identified in the proteomic datasets and browse interactively in the PPI and regulatory networks, ultimately offering a structured entry point for researchers in proteomics, nutrition, and nutraceutical science to investigate the molecular composition of human milk and its potential systemic implications.

## Methods

### Proteomics Data Description

Proteomics datasets of Human milk were mined from ProteomeXchange, ending up with 8 datasets as presented in Supplementary Table 1, which included a total of 1,175 raw human milk samples in the CHILD repository. The largest dataset retrieved was PXD034806, which includes a Liquid Chromatography Mass Spectrometry (LC-MSMS) dataset of 367 human milk samples from the ChildDB. This dataset was initially used to study the proteome relation to allergies of the mother and the allergy development of the infant. Raw data were retrieved for each dataset, and after filtering out additional samples from fractionation protocols (such as in the case of the PXD021974 dataset) and common samples between the PXD036477 and PXD034806 datasets, a total of 753 out of the initial 1,175 raw samples were meta-analysed with the workflow described below.

### Proteomics Data Preprocessing

The MaxQuant/Andromeda [119] v.2.6.6.0 software was selected for preprocessing, with the following parameters: protein false discovery rate (FDR) cutoff of 0.01, minimum peptide length for unspecific search 8 and maximum of 25, variable modifications of acetylation of the peptide N-term, deamidation of the side chains of asparagine and glutamine, and oxidation of methionine, Trypsin/P digestion with a maximum of 2 missed cleavages, and match between runs option activated. The raw data were searched against human proteins retrieved from UniProt (20,421 reviewed UniprotKB/Swiss-Prot proteins as of December 2024). The steps of the preprocessing the quantitative data included the control for strict FDR ratios (5% at peptide and protein levels), filtering proteins with more than 30% missing values across all samples, normalizing against the total ion intensity, and imputing missing values using k-NN impute. Regarding the proteins selected for protein-protein interaction analysis, we applied a more stringent filtering layer before imputation, keeping proteins identified in at least 20% of the samples across all proteomics datasets of human milk.

### Protein Regulatory Network Reconstruction and Analysis

The reconstruction of the protein regulatory networks was conducted with ARACNe-AP algorithm [120, 121] for network inference, complemented by statistical correction for multiple testing, integration of biological directionality through the SIREN method [122]. The pipeline begins with the application of the ARACNe-AP algorithm, which infers regulatory interactions among genes or proteins based on mutual information (MI) [123] computed from protein abundance profiles. To enhance robustness, a bootstrapping approach was employed by performing multiple ARACNe runs with different random seeds. The resulting networks were then aggregated, and a three-step filtering strategy was applied to retain only strong and biologically meaningful interactions. First, a threshold was applied to the aggregated network obtained from the bootstrap process. Second, a dynamic threshold was assigned individually to each protein based on the average strength of its interactions. Third, a global upper-bound threshold defined by ARACNe was applied to further restrict retained interactions. To address the problem of multiple hypothesis testing inherent in large-scale network inference, the Benjamini-Hochberg (BH) procedure [124] was applied and only interactions with corrected p-values below 0.05 were retained for further analysis. Once the corrected undirected network was obtained, biological directionality was inferred using the SIREN method. Directionality scores were calculated and appended to the network, resulting in a directed interaction map in which edges have both magnitude, denoted as MI in the algorithm, and orientation. Finally, to avoid redundancy, duplicate interactions, including bidirectional edges with the same inferred directionality, were evaluated, and among mirrored edges only the one with the stronger directionality score was retained. Self-loops, defined as edges connecting a node to itself, were also removed.

Analyses on the constructed networks were performed using the InSyBio BioNets tool [125], part of the InSyBio Suite (https://suite.insybio.com/), for network metric calculation, clustering using the Markov Clustering (MCL), Restricted Neighborhood Search Clustering (RNSC), and ClusterOne algorithms, as well as network comparison. Custom R and Python scripts were used for downstream analysis and visualization of pathway and functional enrichment results. Pathway enrichment analysis was performed using the DAVID web tool [126], the GTEx portal [127] (https://www.gtexportal.org/) was used to investigate the cell-type specificity of the expression profiles of proteins of interest, and the ChEA3 portal [128] (https://maayanlab.cloud/chea3/) was used for transcription factor enrichment analysis.

### Protein-Protein Interaction Network Reconstruction

The Protein-Protein Interaction (PPI) network reconstruction, based on the human milk proteins identified in the mined human milk MS data, was performed using the predicted interactions from the Evolutionary Optimization Algorithm-based (EOA) methodology suggested by Zaverdas, Stojceski, et al [43], in the context of the taste receptor interactome network reconstruction. This methodology is based on a hybrid combination of an evolutionary heuristic optimization method with machine learning classification and regression methods to develop classification models for the prediction of physical binary positive PPIs based on 61 functional, orthology, sequence, co-expression, and structural features. This method also provides a regression model to estimate PPI binding affinity, and, along with the classifier’s prediction probability, this is used to rank the predicted PPIs.

The network reconstruction was carried out using a list of proteins identified in all the proteomics datasets. This list was reduced by filtering out proteins that were quantified in less than 20% of the samples in all the datasets where they have been identified. Upon filtering, the final reduced list is used for creating binary combinations (PPIs) between them. All the possible combinations (potential binary PPIs) are retrieved from this list, and then the EOA methodology is used for identifying the positive PPIs from the multitude of potential ones, which in turn were used in the network reconstruction phase.

Network visualization was performed with the Cytoscape [129] v.3.10.1 software and network clustering carried out using the Markov Clustering (MCL) [130], Molecular Complex Detection (MCODE) [131], and Leiden [132] algorithms. The parameters for the algorithms were: Granularity 4, 2.5, and 2 for the MCL; for MCODE, haircut and fluff were applied, node score cutoff was set to 0.3, k-core to 2, max depth to 200, and degree coefficient to 3 and 2; and for Leiden, resolution was set to 0.1 and 0.5. Clustering assessment metrics were applied to evaluate the results and choose the optimal clustering algorithm and its parameters in order to evaluate the biological relevance of the findings. Global and intra-cluster metrics were computed by analyzing the clustered subgraph of the network, calculating topology-based measures (e.g., density, degree, clustering coefficient) using NetworkX v.3.4.2 and Cytoscape-exported attributes, and assessing overall cluster separation using Node2Vec embeddings followed by silhouette scoring based on Cytoscape-derived cluster labels. For assessing each cluster in the network, the per-cluster Density, Inertia, and Silhouette Coefficient were calculated by constructing subgraphs for each cluster from the original network. Then, the per cluster sub-graph density was calculated using NetworkX, and node-level topological features (e.g., betweenness, closeness, clustering coefficient) were standardized via z-score normalization.

From the clustering metrics, a final network is chosen based on the Silhouette Coefficient and Density Metrics (for the rationale, see Supplementary Notes 1). For the selected network, a per-cluster Gene Ontology (GO) term and Protein Groups enrichment analysis was performed against all the network’s proteins, in order to find in which biological processes, molecular functions, cellular components, and protein groups each cluster is significantly enriched in regard to the other clusters and genes in the network. GO Term enrichment was also performed in both the full and filtered lists of proteins, which initialized the network creation (Supplementary Fig. 1). For better focus on the biological relevance of the findings, all the graphs created contain clusters and genes with an adjusted p-value of enrichment being < 0.05.

### Molecular Dynamics

To elucidate and characterize the HM-related PPIs identified using the ML-based EOA pipeline, we selected the top interactions from the multitude of positively predicted ones for further Molecular Dynamics (MD) analysis. The selection of the PPI pairs for the MD simulation was based on the following criteria: 1) the involvement of at least one milk-specific protein; 2) Both proteins to have an intrinsic disorder score (calculated using the DisPredict v3.0 [133] software) below 0.5; 3) To be among the top 20 interactions in terms of the average score of the classifier’s probability and the computationally predicted affinity; 4) Both proteins should have PDB structures available and not derived from homology modelling; 5) To limit the computational power required for the simulation, we decided to consider PPIs composed by proteins with fewer than 350 amino acids. Given that L-lactate dehydrogenase B chain (LDBH, UniProt ID: P07195) appeared in 13 of the top 20 PPIs, it was identified as the most recurrent interacting protein. Consequently, we selected LDBH as the fixed anchor and prioritized the three top-ranked PPIs involving this protein. The selected interactors with LDBH were 14–3-3 protein zeta/delta (YWHAZ - Uniprot ID: P63104), nucleoside diphosphate kinase B (NME2 - Uniprot ID: P22392), and cytoplasmic malate dehydrogenase (MDH1 - Uniprot ID: P40925). The atomistic structure of LDBH, YWHAZ, NME2, and MDH1 proteins was collected from the PDB IDs 7DBJ [134], 2O02 [135], 3BBB [136], and 7RM9 [137], respectively.

These three PPI pairs were docked using HDOCKlite v1.1[138, 139] and the first 5 docked poses were selected as starting points for the simulations. Each docked model was placed at the center of a dodecahedral simulation box with a minimum distance of 1.5 nm between periodic images and solvated using TIP3P [140] water molecules. The final solvated systems, following the addition of Na + and Cl− ions to a physiological concentration of 0.15 M, contained between 90,000 and 120,000 particles in total. The Amber19SB [141] force field was used to define protein topology through an all-atom approach, and each system was minimized using the steepest descent method for 5000 steps. We then performed the equilibration procedure through one MD simulation of 500 ps under the NVT ensemble and a subsequent MD simulation of 500 ps under the NPT ensemble. For the equilibration protocol, the v-rescaling [142] temperature coupling algorithm with a time constant of 1.0 ps was applied to keep the temperature at 310 K. C-rescale [143] semi-isotropic pressure coupling algorithm with a reference pressure of 1 bar and a time constant of 5.0 ps was employed. Then, all systems were simulated for the production run in the NPT ensemble with 4 fs time steps [144], using v-rescaling thermostat and c-rescale barostat, with a time constant of 1.0 ps and 5.0 ps, respectively. Each starting pose was simulated for 200 ns in 3 replicas, for a total of 9 μs of sampled time. Electrostatic interactions were calculated by applying the particle-mesh Ewald [145] (PME) method and van der Waals interactions [146] were defined within a cut-off of 1.0 nm. A time step of 4 fs was achieved through the application of a hydrogen mass repartitioning scheme[144], in which the minimum atomic mass was scaled by a factor of 3, in combination with LINCS constraints[147]. Trajectories were collected every 20 ps and the Visual Molecular Dynamics [148] (VMD) package was employed to visually inspect the simulated systems. GROMACS 2025 [149, 150] package was used for simulations and data analysis. The last 20 ns of the 200 ns production run of each simulation were considered for the analyses.

### Web platform of Proteomic Atlas of Human Milk

The application utilizes PHP v7.4.33 as the backend programming language, built on the Yii v1.1.23 framework, a straightforward framework compatible with legacy systems. Networks were reconstructed using the Cytoscape software as discussed in section 2.4 and integrated using its dedicated JSON format (cyjs) designed for web-based visualization. The front-end was developed using JavaScript for interactivity, with HTML5 and CSS managing the structure and styling. PostgreSQL v16.9 serves as the database management system.

## Supplementary Material

Supplementary Files

This is a list of supplementary files associated with this preprint. Click to download.

• HumanMilkManuscriptSuppInfoNatFoodfinal.docx

## Data Availability

All data generated and analyzed in this study, as well as the networks in an interactive form, are available through the Human Milk Proteomic Atlas web interface and can be accessed at: http://atlas-galatea.insybio.com/.

## Code Availability

All the proteomics and network data generated in this study are publicly available in the web application created for this purpose as described in the Data Availability section (Human Milk Proteomic Atlas web interface).
